# Genopathomic profiling identifies signatures for immunotherapy response of lung adenocarcinoma via confounder-aware representation learning

**DOI:** 10.1016/j.isci.2022.105382

**Published:** 2022-10-17

**Authors:** Jiajun Deng, Jiancheng Yang, Likun Hou, Junqi Wu, Yi He, Mengmeng Zhao, Bingbing Ni, Donglai Wei, Hanspeter Pfister, Caicun Zhou, Tao Jiang, Yunlang She, Chunyan Wu, Chang Chen

**Affiliations:** 1 Department of Thoracic Surgery, Shanghai Pulmonary Hospital, School of Medicine, Tongji University, Shanghai, P.R. China; 2 Shanghai Jiao Tong University, Shanghai, P.R. China; 3 MoE Key Lab of Artificial Intelligence, AI Institute, Shanghai Jiao Tong University, Shanghai, P.R. China; 4 Dianei Technology, Shanghai, P.R. China; 5 Department of Pathology, Shanghai Pulmonary Hospital, School of Medicine, Tongji University, Shanghai, P.R. China; 6 Huawei Hisilicon, Shanghai, P.R. China; 7 Harvard University, Cambridge, MA, USA; 8 Department of Medical Oncology, Shanghai Pulmonary Hospital, School of Medicine, Tongji University, Shanghai, P.R. China; 9 The First Hospital of Lanzhou University, Gansu, P.R. China; 10 The International Science and Technology Cooperation Base for Development and Application of Key Technologies in Thoracic Surgery, Gansu, P.R. China

**Keywords:** Immunology, Cancer, Artificial intelligence

## Abstract

Immunotherapy shows durable response but only in a subset of patients, and test for predictive biomarkers requires procedures in addition to routine workflow. We proposed a confounder-aware representation learning-based system, genopathomic biomarker for immunotherapy response (PITER), that uses only diagnosis-acquired hematoxylin-eosin (H&E)-stained pathological slides by leveraging histopathological and genetic characteristics to identify candidates for immunotherapy. PITER was generated and tested with three datasets containing 1944 slides of 1239 patients. PITER was found to be a useful biomarker to identify patients of lung adenocarcinoma with both favorable progression-free and overall survival in the immunotherapy cohort (p < 0.05). PITER was significantly associated with pathways involved in active cell division and a more immune activating microenvironment, which indicated the biological basis in identifying patients with favorable outcome of immunotherapy. Thus, PITER may be a potential biomarker to identify patients of lung adenocarcinoma with a good response to immunotherapy, and potentially provide precise treatment.

## Highlights

• Genetic profile could be extracted by deep learning from digital histologic slides
• Extracted pathomic signature could identify patients’ response to immunotherapy
• The extracted pathomic signature was involved in an active immune microenvironment

## Graphical abstract

**Figure:**
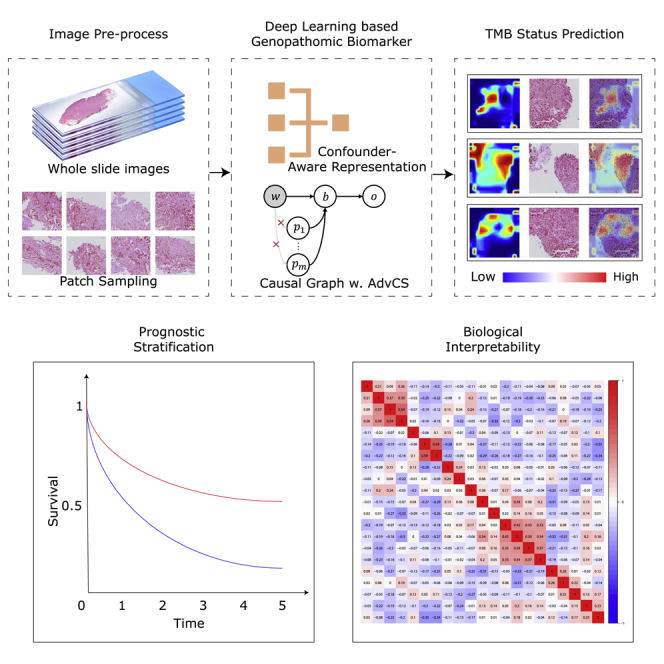

## Introduction

Immune checkpoint inhibitors (ICIs) (Ribas and Wolchok, 2018), as an emerging antitumor therapy, are gradually rewriting clinical treatment paradigms for a variety of cancers, including non-small-cell lung cancer (NSCLC) (Ferris et al., 2016; Borghaei et al., 2015). In patients with advanced NSCLC, immunotherapy was shown to improve the clinical outcomes in previous clinical trials, but only in a subset of patients (Herbst et al., 2016; Borghaei et al., 2015). Therefore, identifying suitable predictive biomarkers for immunotherapy is warranted and has become an urgent need for the application of tumor immunotherapy (Hegde and Chen, 2020). Previous studies have highlighted the strong relationship between the tumor mutational burden (TMB) and the activity of ICI therapies across multiple types of cancers (Rizvi et al., 2015; Marabelle et al., 2020). TMB is determined by whole exome sequencing (WES) and is defined as the total number of coding and somatic mutations (Rizvi et al., 2015). Such tests face barriers to being deployed in routine oncology workflows due to the high turnaround time, complexity, and cost (Chan et al., 2019).

Genetic variation in tumor cells causes functional changes, resulting in tumor cell morphological changes (Schmauch et al., 2020). Such morphological changes can be seen in histological images, which are the most common modality in the diagnostic workflow. With the development of artificial intelligence, comprehensive imaging analyses of histological slides using computational approaches could reveal previously concealed information and decode malignant disease progression and prognosis (Bera et al., 2019). Recent studies revealed that the extracted features from digitized pathological images were clinically related to molecular features and showed predictive power for genetic profiles (Coudray et al., 2018). These pathomic studies allow for the correlation between histopathological characteristics and molecular and genetic alterations, thereby improving disease depiction.

**Figure 1:**
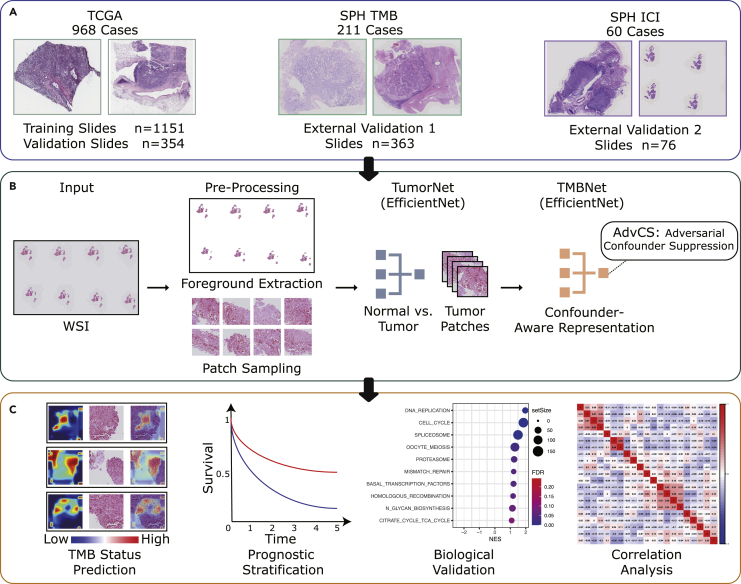
Schematic representation of the study design (A) Whole-slide images (WSI) and corresponding clinical records and available genetic profiles from TCGA were used for the training and for the internally validation of the dataset; two external datasets were included to independently evaluate the proposed framework. (B) The proposed PITER framework was designed to predict the status of the tumor mutational burden (TMB) from WSIs, which served as a biomarker for the immunotherapy response in a subsequent correlation analysis with genetic and immune infiltration data. The whole pipeline consists of 2 deep neural networks based on EfficientNet, TumorNet to classify tumor/normal patches, and TMBNet to classify the TMB status. We propose an adversarial confounder suppression (AdvCS) technique to eliminate the influence of confounding variables. (C) The developed PITER was applied to predict TMB status and prognosis following immunotherapy. Genomics and transcriptomics data were used to indicate the potential interpretation of the PITER. TCGA, The Cancer Genome Atlas. PITER, the genopathomic biomarker for immunotherapy response.

Deep learning models have benefited from the development of both software and hardware techniques and have shown professional-level performance in the context of medical imaging analysis tasks, including the classification of dermoscopy images (Esteva et al., 2017), chest computed tomography (Ardila et al., 2019), and histological slides (Campanella et al., 2019). Moreover, deep learning models have been shown to be promising for assisting with whole-slide imaging (WSI) analyses, including genetic mutation identification (Fu et al., 2020), microsatellite instability status determination (Kather et al., 2019b), and prognosis (Kather et al., 2019a) prediction. Deep learning algorithms have been proven to successfully predict the level of TMB by using WSI (Jain and Massoud, 2020). However, few prior studies have developed biomarkers validated in an immunotherapy cohort. In this study, we developed a genopathomic biomarker for immunotherapy response (PITER) by leveraging histopathological and genetic (i.e., TMB) characteristics with deep learning techniques. By building a causal graph of this modeling procedure, we identified the confounding variables that were prone to overfitting and further designed an *adversarial confounder suppression* (AdvCS) scheme to improve learning performance. The AdvCS technique was easy to implement and significantly stabilized the training procedure. The proposed PITER was first developed and validated using data from the multicenter The Cancer Genome Atlas (TCGA) database to predict TMB from WSIs. We then independently validated our method in external cohorts in terms of TMB predictive performance and ICI efficacy assessment (Figure 1). Finally, we illustrated the underlying association between the PITER prediction and ICI response by analyzing genetic data.

## Results

### Development of the genopathomic biomarker (PITER)

We first built the TumorNet to classify lung cancer tissue in 20% of randomly selected patients. After training, the accuracy of the TumorNet to classify normal, lung adenocarcinoma, and squamous cell carcinoma in the TCGA dataset was 0.96. We then tested the discrimination ability on our SPH TMB dataset, revealing an accuracy of 0.81 for classifying two malignancies (Figure S1).

**Figure 2:**
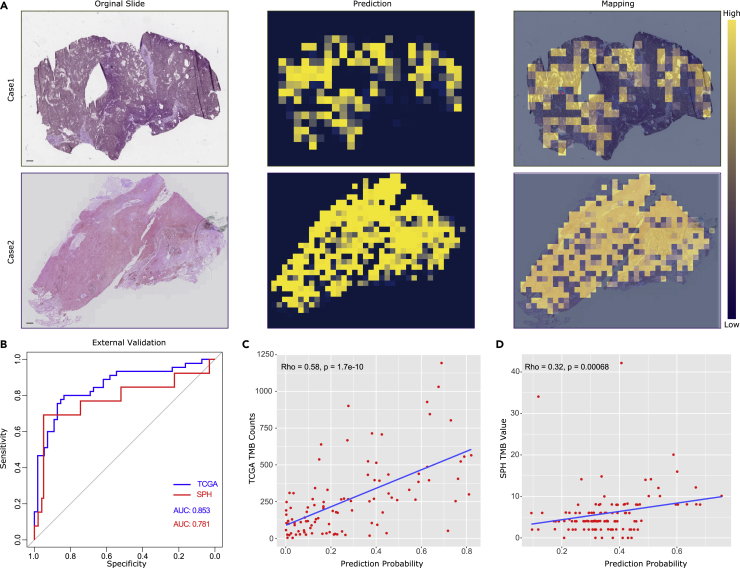
TMB prediction performance of the proposed biomarker (A) Original histological slides and heatmaps of the prediction output. (B–D) Independent validation of TMB prediction performance by the PITER in the TCGA (blue) and SPH (red) validation datasets. Scatterplots of changes in tumor mutational burden value according to probability output from the PITER in the TCGA (C) and SPH (D) validation datasets. TCGA, the cancer genome atlas. AUC, area under curve. TMB, tumor mutational burden.

Next, we trained the TMBNet separately for LUAD and LUSC. Representative cases for the output of the TMBNet are shown in Figure 2A. We internally validated the TMB predictive performance on the TCGA LUAD validation dataset (100 patients with 195 slides), which was unseen during the training process (Table S1). For LUAD (Figure 2B), the areas under the receiver operating characteristic curve (AUCs) for the PITER to stratify high- versus low-level TMB were 0.853 (95% confidence interval [CI]: 0.775–0.932) in the TCGA validation dataset. The PITER score was significantly correlated with the actual TMB value as a continuous variable (Spearman’s rho = 0.58, p < 0.01, Figure 2C). We then evaluated the predictive value of TMB status on an external cohort of 111 patients with 175 slides (Table S2). The AUC reached 0.781 (95% CI: 0.610–0.952) in the SPH TMB dataset, the predictive score output by the PITER was higher in the high-TMB group than in the low-TMB group, and the PITER score was significantly associated with the actual TMB value (Spearman’s rho = 032, p < 0.01, Figure 2D).

For LUSC (Figure S2), the AUC for the proposed method for stratifying high- and low-level TMB was 0.607 (95%CI: 0.595–0.720) in the TCGA LUSC validation dataset (100 patients with 175 slides, Table S3) and 0.524 (95% CI: 0.386–0.663) in the SPH TMB dataset (100 patients with 188 slides, Table S4). As the predictive ability of the proposed method using LUSC cases failed to demonstrate robustness and efficacy in both independent validation datasets, we continued with the framework built on LUAD for the subsequent experiments.

### Predicting survival by PITER in the immunotherapy dataset

**Figure 3:**
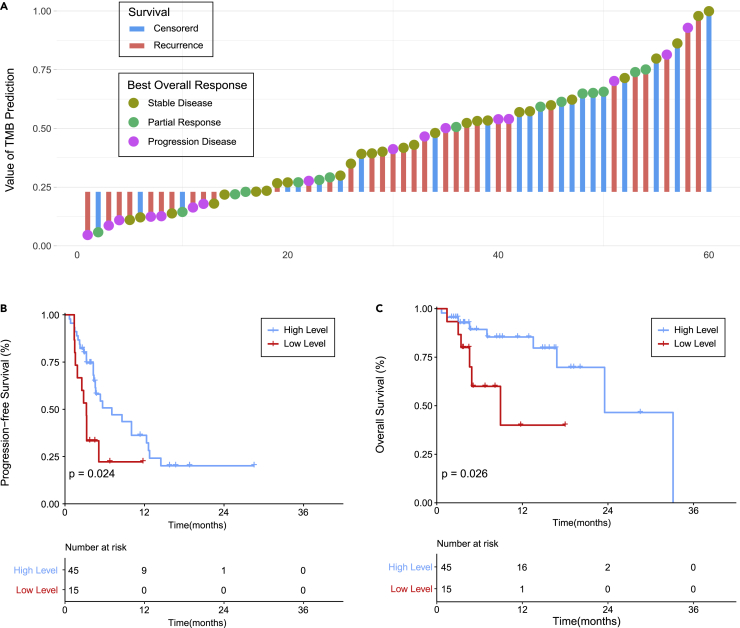
Survival outcomes of the immunotherapy dataset (A) Individual clinical outcomes at the patient level (bars correspond to survival events and dots correspond to the best overall response). (B) Kaplan-Meier curve of progression-free survival stratified by the PITER (high and low levels indicate the probability of treatment response). (C) Kaplan-Meier curve of overall survival stratified by the PITER (high and low levels indicate the probability of treatment response).

We applied the same cutoff point (cutoff value: 0.23, Table S5) in the immunotherapy treatment group for further prognostic analysis to test the prognostic performance of the PITER in both overall survival (OS) and progression-free survival (PFS). Based on the PITER, patients can be divided into two groups with significantly different prognoses. There was no significant difference between the two groups based on the clinical and pathological variables except for sex. The survival curves analysis showed that the group predicted to have a high level of TMB demonstrated a significant survival benefit over the group predicted to have a low level of TMB in both PFS and OS among the entire cohort (Figure 3). In the multivariable Cox analysis, the PITER score remained significant after adjusting for other clinical and pathological variables in the analysis of both the PFS (hazard ratio [HR]: 0.44, 95% CI: 0.21–0.92, p = 0.03) and OS (HR: 0.32, 95% CI: 0,10–0.99, p = 0.04) (Table S6).

### Biological validation of the PITER

**Figure 4:**
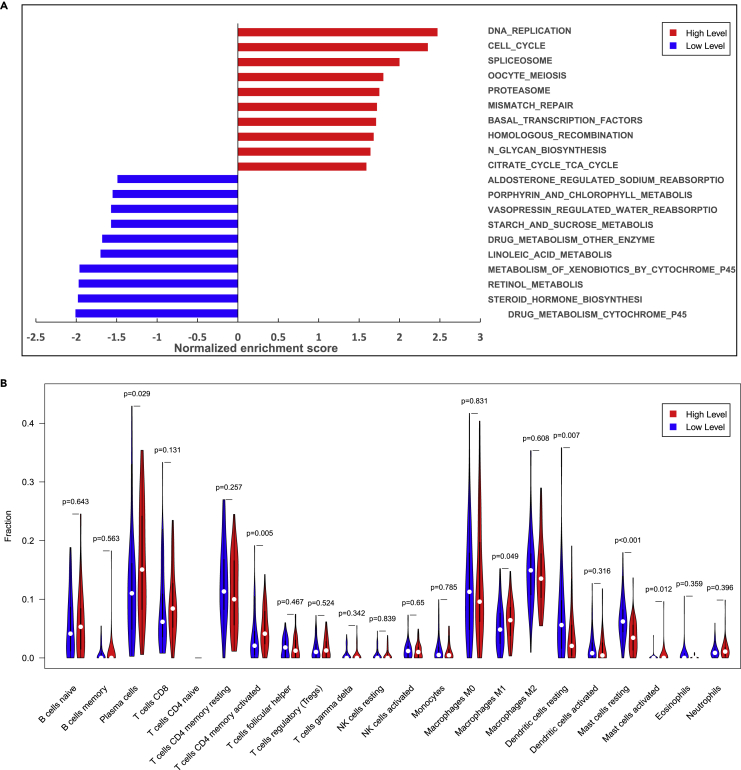
Biological validation (A) Association of the PITER and underlying pathways found by gene enrichment analysis. (B) Variance in the immune microenvironment between groups with different immunotherapy response probabilities. High and low levels indicate the probability of treatment response.

To validate the biological basis of the PITER, we evaluated the TCGA cohort with the matched copy nucleoid variation data available (Figure S3). Through ranked GSEA, we found that the top gene sets that showed a significant association with the biomarker were involved in the tumor cell cycle, DNA mismatch repair, and homologous recombination repair pathways (Figure 4A). This indicates that higher TMB correlates with more active cell division and frequent DNA replication, suggesting a higher degree of malignancy. The difference in this pathway reflects the malignancy of the tumors to a certain extent. The higher the degree of tumor malignancy, the stronger the replication and DNA repair ability as well as the higher the TMB value. In contrast, in tumors with a higher degree of malignancy, the genes affected in the low-TMB group were more related to cell metabolism, which also indicates that the frequency of gene replication and mutation is higher in the high-TMB group.

To further investigate the association between biomarkers and the tumor-immune microenvironment, we next deconvoluted TCGA RNA sequencing data to infer the tumor-immune microenvironment (Figure 4B). Differences were found in plasma cells, T cells (memory CD4), dendritic cells, and mast cells. The correlation heatmap showed correlations between CD4 T cells and CD8 T cells as well as plasma cells and naive B cells (Figure S4), indicating that a link between immune cells and improved treatment response may exist and provides an explanation for the immunotherapy therapeutic decision for PITER-predicted TMB-high cancers.

### Visualization of PITER prediction

**Figure 5:**
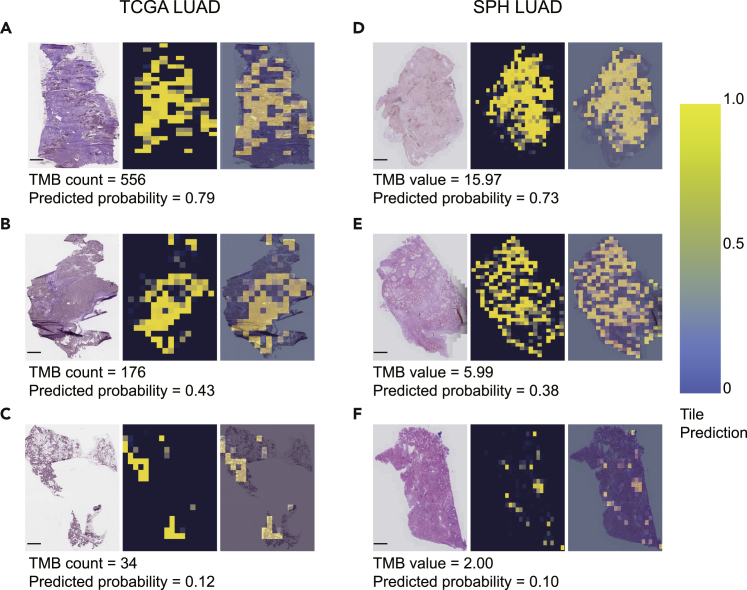
Visualization heatmaps of the histology slides used for prediction in six randomly selected patients from the TCGA (left panel) and SPH (right panel) validation datasets at three levels of TMB status (A–C) Cases with varying TMB counts from high to low of TCGA-LUAD cohort. (D–F) Cases with varying TMB values from high to low of SPH-LUAD cohort. TCGA, the cancer genome atlas. LUAD, lung adenocarcinoma. TMB, tumor mutational burden.

To further examine the relationship between WSI and TMB, we reviewed the activation heatmap of the biomarkers for further investigation. The PITER provides a visualization of the spatial and intensity distribution of TMB predictions in different cohorts for each digital slide (Figure 5). Yellow areas of the heatmap indicate regions of the tissue predicted by the algorithm to have high TMB. The pattern of the predicted region was similar across different slides at the same level of TMB, even though the slides from different cohorts were not processed in the same manner. The PITER predicted regions of both low- and high-TMB within the boundary of the tumors, suggesting that the PITER may be able to detect morphological associates of TMB that are heterogeneous across the cancer.

## Discussion

The advent of immunotherapy has completely changed the treatment prospects of NSCLC (Ferris et al., 2016; Borghaei et al., 2015). However, studies have found that the objective response rate to immunotherapy remains relatively low (Herbst et al., 2016; Borghaei et al., 2015). The development of new predictive markers to determine whether patients will benefit from immunotherapy is of the utmost importance. In this study, we proposed a genopathomic biomarker, the PITER, to study TMB status using digital histology slides. The proposed PITER robustly and consistently predicted the TMB status of patients with lung adenocarcinoma in both internal and external validation cohorts. Our results showed that the PITER was correlated with the response to immunotherapy in an independent lung adenocarcinoma patient cohort. The biological basis was evaluated through a gene expression analysis of biological pathways.

As a genetic disease, the neoplastic transformation of malignancy is the product of the accumulation of somatic mutations in affected cells, which could be revealed by morphological changes detectable in routine histological slides by using a state-of-the-art deep learning approach. H&E imaging resources have mainly been used to certify histological subtypes and diagnoses with some restricted extraction of standard histopathological variables, such as grade, mitotic activity, and lymphocytic infiltrates (Cooper et al., 2018). Digital pathology allows us to decipher pathomic links by applying computational methods to extract more sophisticated features and ultimately correlate them with molecular and genetic findings. The PITER enables the in-depth characterization of the tumor TMB phenotype and provides a deeper understanding of cancer biology to better aid clinical decisions. TMB is an indicator of the genetic variation profile of a tumor. Several researchers have successfully developed artificial intelligence models to predict the TMB status in bladder cancer (Xu et al., 2019), gastric cancer (Kather et al., 2019b), and lung adenocarcinoma (Jain and Massoud, 2020). However, due to the lack of a publicly available dataset, previous research has not further validated their models’ predictive capability in an external dataset or their predictive value for response to immunotherapy. Previously, Jain et al. proposed a deep learning approach to predict the level of TMB by aggregating three magnifications (X5 AUC of 0.72, X10 AUC of 0.80, and X20 AUC of 0.81) and achieved an AUC of 0.92 (Jain and Massoud, 2020). In this study, the PITER was trained and tested using 10× magnification. The PITER achieved a comparative performance when predicting TMB status when compared to the performance relying on single magnification reported by Jain et al. Importantly, the robustness of the TMB evaluation performance was tested on FFPE tissue from an independent external validation cohort. In our experiments, the simple aggregation of multiple magnifications (Jain and Massoud, 2020) did not improve the performance of the model on the external validation cohorts. The application of a low-magnification approach significantly reduces the computational costs. On the other hand, the smaller the required magnification, the more likely it is that the approach will be deployed in a clinical workflow.

The predictive value of the PITER and response to immunotherapy was validated in an independent immunotherapy cohort in our study. Previous studies have shown the predictive value of artificially intelligent biomarkers based on other modalities, such as radiological (Sun et al., 2018) and genetic features (Cristescu et al., 2018), in patients with advanced NSCLC, but not using digital histology slides. Based on the pathology images, the PITER was consistently shown to be a prognostic factor for both the OS and the PFS rate of the patients enrolled in the study. Previous lung cancer prognostic models were developed using clinical data. In this study, we showed that the PITER has the predictive power to stratify patients with significant differences after adjusting for clinical variables. Furthermore, our results revealed that the PITER was specific for immunotherapy response prediction in advanced lung adenocarcinoma, but not in patients treated with various other strategies (Figure S3). TMB has been shown to be predictive of immunotherapy response and has become a useful biomarker among many tumor types for identifying potential patients who will benefit from immunotherapy (Rizvi et al., 2015; Hugo et al., 2016). However, not all patients are screened for TMB status outside of high-volume tertiary care centers. Although the PITER was trained by using TCGA, which consisted of slides from various registries, its generalizability was tested through external validation using FFPE tissue slides (Figure 1A) from our institution with available TMB data (surgical resection) and survival data (surgical resection and small diagnostic biopsy specimens); the results were consistent with those of the previous study (Coudray et al., 2018). Based on these results, it is prudent to point out that the proposed PITER corresponding to genetic variation has the potential to predict immunotherapy response. As pathology imaging is a routinely acquired diagnostic modality in the clinical management of tumor patients, our model could therefore easily facilitate the determination of lung adenocarcinoma patient TMB status and facilitate access to immunotherapies for a larger number of eligible patients. This finding is consistent with previous reports regarding the application of a deep learning approach for predicting the level of TMB.

Deep learning for image recognition automatically learns internal representations end-to-end. The internal transcriptomic representation learned by the PITER during the prediction of RNA sequencing data may constitute an important step toward understanding the biological descriptors required for solving clinical classification problems and understanding the link between the tissue- and molecular-level information. Previous studies have shown that deep learning models capture morphometric features and complex structures in tissue images that represent specific phenotypes (Yoo et al., 2020). Gene enrichment analyses have shown associations between the PITER with DNA replication and the proliferative gene set, indicating potential factors that impact tumor immunogenicity and enhance the immune response. Sensitivity to immunotherapy is based on not only intrinsic tumor factors but also the complex interplay between cancer and its microenvironment. Previous studies have reported that several immunogenic microenvironment factors, such as T cells (CD4^+^) (Du et al., 2018), macrophages (Zappasodi et al., 2018), and mast cells (Somasundaram et al., 2021), are prognostic and predictive markers in many tumor types. Through the analysis of the TCGA RNA sequencing data, we found that the PITER could stratify patients with different tumor-immune microenvironments. It was found that the cellular components that promote immune activation increased significantly in the high-TMB group, while the low-level TMB markers represented immunosuppressed, thereby revealing that the PITER could learn features of the tumor-immune microenvironment from histological images.

In conclusion, the PITER, a clinically useful prognostic biomarker, was developed using a deep learning approach to digital pathology whole-slide analysis. As evaluated in multiple cohorts and independent populations, the PITER could be an efficient and reliable method for evaluating the TMB statuses of patients with lung adenocarcinoma as well as their likely responses to immunotherapy. These findings indicate the potential for the PITER to be used by clinicians to improve decision-making related to immunotherapy choices beyond whole exome sequencing.

### Limitations of the study

Several limitations of our study should be acknowledged. Although immunotherapy response prediction was evaluated externally, only Chinese patients with advanced lung adenocarcinoma from a single institution were included. Further validating the present findings with patients of other ethnicities requires a prospective and multi-institutional study with a large cohort. In addition, the study used TMB as the only label to build the predictive model, and the model’s efficacy may be limited because TMB is not a perfect predictor for patient response to immunotherapy treatment. Thus, a joint analysis of more comprehensive labels, including tumor microenvironment-specific genetic alterations, should be performed. In addition, the transcriptomic and proteomic data could provide useful insights and increase the overall performance of our model. Furthermore, the proposed method seemed to be effective only for LUAD; one possible reason for this is that the level of disease progression was not associated with morphological changes in LUSC. Additional attempts could be focused on functional staining of the tumor microenvironment. Moreover, although the proposed AdvCS technique was motivated by the analysis of causal graph, this study was indeed about correlation analysis. Causality in clinical applications is difficult to pursue, as there are too many known or unknown factors in the real world. Nevertheless, by introducing more medical confounders into the optimization procedure of representation learning (Zhao et al., 2020), it is possible to further improve model predictive performance. Finally, only lung cancer was included in this study, and the generalizability of the PITER to other cancer types or subtypes was unable to be validated. Previous studies have shown the ability to transfer the learning of similar computational models to generalize the approach for different types of cancer (Lu et al., 2021). In the future, the performance of this model may improve considerably using larger, richer datasets for training.

## STAR★Methods

### Key resources table

**Table:** 

REAGENT or RESOURCE	SOURCE	IDENTIFIER
**Software and algorithms**		

GenoPathomic biomarker of ImmunoThErapy Response	This Paper	https://github.com/NotF404/TCGA_TMB
EffficientNet	(Tan and Le, 2019)	https://arxiv.org/pdf/1905.11946.pdf
PyTorch	(Paszke et al., 2019a)	https://arxiv.org/pdf/1912.01703.pdf
Python	Python Software Foundation	https://www.python.org/
R software	The R Foundation	https://www.r-project.org/

**Other**		

Lung adenocarcinoma tumor mutational burden dataset	The Cancer Genome Atlas	https://portal.gdc.cancer.gov/
Lung squamous cell carcinoma tumor mutational burden dataset	The Cancer Genome Atlas	https://portal.gdc.cancer.gov/

### Resource availability

#### Lead contact

Further information and requests for resources and reagents should be directed to and will be fulfilled by the lead contact, Chang Chen (changchenc@tongji.edu.cn).

#### Materials availability

This study did not generate new unique reagents.

### Experimental model and subject details

#### Study design and patient cohorts

In this multicohort study, a deep learning analysis was applied retrospectively to four independent cohorts of patients with NSCLC (Figure S5). The TMB phenotype datasets from the TCGA Lung Adenocarcinoma (LUAD) and Lung Squamous Cell Carcinoma (LUSC) datasets were included on August 14, 2020 (https://portal.gdc.cancer.gov). We collected a total of 1006 patients with whole exome sequencing (WES) data and 1505 corresponding digital histological images to generate the training and validation data for the development of the PITER, the proposed genopathomic biomarker.

After training and internally validating the proposed framework, we included 211 patients with 410 slides diagnosed with LUAD and LUSC between 2012 and 2015 (the Shanghai Pulmonary Hospital [SPH, Shanghai, China] TMB dataset) to externally validate the TMB prediction ability of the biomarker. In addition, to further evaluate the predictive value of PITER in identifying how patients with advanced lung adenocarcinoma benefit from immunotherapy, an SPH immunotherapy dataset including patients with 60 unresectable and metastatic lung adenocarcinoma who had received ICI therapy between 2015 and 2018 was included (He et al., 2020). The WSI data aligned with the genetic and clinical characteristics retrieved from SPH. Further details of data acquisition and process are provided in the supplemental information. Our retrospective study was approved by SPH’s institutional review board (Reference Number: L20-333-1), and informed consent was waived.

#### Details of data acquisition and process

In the Cancer Genome Atlas (TCGA) LUAD and LUSC datasets, whole exome sequencing (WES) data with corresponding images were used to generate the training and validation data of the PITER, allowing for the estimation of the level of tumor mutational burden (TMB) using WES data and a deep learning approach to the analysis of the corresponding images of the tumor samples. The TMB data of the TCGA patients were computed as the total number of nonsynonymous mutations in the WES of the patient’s cancer by applying VarScan2 (Koboldt et al., 2012). The cutoff value used to stratify patients as TMB-high or TMB-low was 206, as it this number represented approximately 10 mutations per megabase (mut/Mb) when analyzing the genetic profile established in the FoundationOne CDX panel, according to a previous study (Jain and Massoud, 2020).

The SPH TMB dataset was used to externally validate the performance of the genopathomic biomarker for the immunotherapy response (PITER) on differentiating TMB-high and TMB-low. This validation dataset included patients who had available TMB data and were treated by surgical resection for lung adenocarcinoma and squamous cell carcinoma. The details of the data processing and WES are reported in our previous study (Jiang et al., 2019). Consistent with a previous clinical trial (Hellmann et al., 2018), TMB-high was defined as TMB value ≥10 mut/Mb; otherwise, tumors were defined as TMB-low. The SPH immunotherapy dataset, consisted of patients with advanced lung cancer enrolled in a phase 1 trial, was used to evaluate anti-PD-1 or anti-PD-L1 monotherapy at our institution. This dataset was used to infer the association between the PITER and clinical response in accordance with the Response Evaluation Criteria in Solid Tumors (RECIST) version 1·1 (Eisenhauer et al., 2009) and the progression-free survival (PFS) and overall survival (OS) rates. In these datasets, WSI data were available for the TCGA datasets, formalin-fixed paraffin-embedded (FFPE) samples from the SPH TMB dataset, and both FFPE samples and tumor biopsy samples from the SPH immunotherapy dataset. WSI data from the SPH dataset were scanned with Motic EasyScan at 40× equivalent magnification and stored in SVS format.

### Method details

#### Development of deep learning genopathomic biomarker

As depicted in Figure 1B, several steps were taken in the development of the PITER to obtain the final outputs. The WSIs were first preprocessed into stain-normalized patches of 512×512 from the extracted foreground. All tissue slides were processed into patches, for the patients with single or multiple tissue slides, the slide(s) from a patient are processed into a bag of patches for subsequent analysis. The patches were further classified as normal or tumor using the deep neural network TumorNet. We then used another neural network, TMBNet, to predict the TMB level (high or low) according to the patient-level TMB label, where only tumor patches were used. However, we observed that TMBNet was prone to overfitting. By analyzing the causal graph of this modeling procedure, we found that one of the main causes of overfitting was the existence of confounding variables. To address this, an AdvCS technique was implemented to improve the performance of TMBNet. The patch-level predictions were aggregated into WSI-level predictions by calculating the median values. We regarded TMB prediction as a biomarker for immunotherapy response and validated its predictive performance for ICI efficacy. Both TumorNet and TMBNet were developed based on EffficientNet (Tan and Le, 2019), a state-of-the-art convolutional neural network.

#### Causal graph for the PITER

Causality is important in medical imaging (Castro et al., 2020), and causal graphs are a vital tool. To better understand what hinders the model performance in the pipeline, we illustrate the causal graph of the PITER deep learning procedure (Figure S6). In this study, we assumed that TMB, as a biomarker for immunotherapy response, was predictable using WSIs, which could be represented by the ideal causal graph in the left part of Figure S6. However, WSIs were too large to process with limited GPU memory. In practice, it was necessary to transform a WSI into patches before feeding these patches into a neural network, which produced the causal graph shown in the middle part of Figure S6. In this case, WSI became a confounding variable (confounder) when inferring TMB (biomarker) from patches. The confounder could be a shortcut when training TMBNet. As evidenced, we observed that it was easy to classify the slide ID of a patch using the representation of the final layer. In other words, the existence of confounders was a major cause of the overfitting issue.

To address this issue, we proposed an AdvCS technique that encouraged TMBNet to learn in a confounder-aware manner regardless of the whole-slide ID. This technique was implemented with a gradient reversal layer (Ganin et al., 2016) jointly trained to classify the primary slide ID (confounder classifier). The confounder classifier was learned to minimize the cross entropy between its output and slide-ID while its reversed gradient made deep representations of the main task neural network less variant to confounders, thereby reducing the effect of the confounding factors on the learning process (illustrated in the right part of Figure S6). The AdvCS technique was easy to implement and significantly stabilized the training procedure.

In order to verify the usefulness of specific designs in this study, we compared the model performance plain backbones (Inception-V3 (Szegedy et al., 2016) vs. EfficientNet) equipped with stain normalization and the proposed AdvCS technique on the TCGA LUAD dataset. As depicted in Table S7, all compared designs were effective. Thus, we used the EfficientNet with stain normalization and AdvCS for the further analysis.

In TumorNet and TMBNet, the backbones of EfficientNet-B0 (Tan and Le, 2019) pre-trained on ImageNet (Krizhevsky et al., 2017) were trained to minimize the cross-entropy loss between network outputs and patch labels (i.e., tumor or not for TumorNet and high or low TMB for TMBNet). In each batch, 32 patches with a size of 512 × 512 were randomly sampled at the 10× magnification in the tissue region of a WSI. Adam (Kingma and Ba, 2015) with cosine learning rate schedule (Loshchilov and Hutter, 2017) decreasing from 6 × 10-4 to 6 × 10-6 in 20 epochs was used as an optimizer. Weight decay with a factor of 0.01 was adopted to prevent overfitting. We also applied data augmentation strategies, including random rotations of 90 degrees and flipping with a probability of 0.5, to improve the model’s generalization performance. All our experiments were conducted on 4 NVIDIA 2080ti GPUs, and the code was implemented with PyTorch (Paszke et al., 2019b) 1.3.0 and Python 3.7.

#### Gene expression analysis and tumor immune microenvironment investigation

We performed a gene set enrichment analysis to explore the potential genetic pathways related to the PITER for TMB prediction. The RNA-sequencing expression data in fragments per kilobase of transcript per million mapped reads (FPKM) values for all samples were obtained from the TCGA portal and transformed to log2 (FPKM+0.1). A rank of these genes was then created using a fold change approach, which was determined in the different values of the mean gene expression between the high- and low-TMB groups. For the gene set analysis, a preranked gene set enrichment analysis (GSEA) was conducted using the PIANO R package, and expert-curated pathways were tested from the KEGG gene set version 7.2, available from the Molecular Signature Database. A p value less than 0.05 and a false discovery rate (FDR)-p less than 0.25 were applied as the filter conditions for gene pathway enrichment. To investigate the tumor immune infiltration between the high- and low-TMB groups identified by the PITER, CIBERSORT was used to deconvolute the relative proportion of 22 human immune cell phenotypes, including T cells, B cells, NK cells, macrophages, DC cells, and mast cells from the RNA-sequencing data obtained from the TCGA database. After annotating the gene matrix with standard annotation, we applied the CIBERSORT algorithm with R and set P < 0.05 as the cutoff value for statistical significance.

### Quantification and statistical analysis

Comparisons of variables were performed using Student’s t-test for continuous variables as well as the Pearson chi-squared test and Fisher’s exact test for categorical variables. The PITER’s predictive power was evaluated by the area under the receiver operating characteristic curve. The cut-off value for separating the high- and low-TMB groups was obtained from the Youden Index in the training cohort. Confidence intervals were computed based on the percentiles of 10,000 bootstrap replicates. Progression-free survival (PFS) and overall survival (OS) rates were computed using the Kaplan-Meier method and Cox proportional hazards survival estimates. End points were the date of death from any cause for the OS rate and the date of any recurrence or death for the PFS rate. The multivariable analysis included the clinically relevant variables that were significant in the univariable analysis. Statistical analyses were performed using R software V.4.0.2 (http://www.r-project.org/). P < 0.05 was considered to be statistically significant.

## Data Availability

• Image and genetic data of The Cancer Genome Atlas are publicly available as of the date of publication. DOIs is listed in the key resources table. Data regarding TMB and ICI validation datasets from Shanghai Pulmonary Hospital are available from the corresponding author (Prof. Chang Chen via changchenc@tongji.edu.cn) on reasonable requests. • All original code has been deposited at GitHub and is publicly available as of the date of publication. DOIs are listed in the key resources table. • Any additional information required to reanalyze the data reported in this paper is available from the lead contact upon request.

## References

[bib1] Ardila D., Kiraly A.P., Bharadwaj S., Choi B., Reicher J.J., Peng L., Tse D., Etemadi M., Ye W., Corrado G. (2019). End-to-end lung cancer screening with three-dimensional deep learning on low-dose chest computed tomography. Nat. Med..

[bib2] Bera K., Schalper K.A., Rimm D.L., Velcheti V., Madabhushi A. (2019). Artificial intelligence in digital pathology - new tools for diagnosis and precision oncology. Nat. Rev. Clin. Oncol..

[bib3] Borghaei H., Paz-Ares L., Horn L., Spigel D.R., Steins M., Ready N.E., Chow L.Q., Vokes E.E., Felip E., Holgado E. (2015). Nivolumab versus docetaxel in advanced nonsquamous non-small-cell lung cancer. N. Engl. J. Med..

[bib4] Campanella G., Hanna M.G., Geneslaw L., Miraflor A., Werneck Krauss Silva V., Busam K.J., Brogi E., Reuter V.E., Klimstra D.S., Fuchs T.J. (2019). Clinical-grade computational pathology using weakly supervised deep learning on whole slide images. Nat. Med..

[bib5] Castro D.C., Walker I., Glocker B. (2020). Causality matters in medical imaging. Nat. Commun..

[bib6] Chan T.A., Yarchoan M., Jaffee E., Swanton C., Quezada S.A., Stenzinger A., Peters S. (2019). Development of tumor mutation burden as an immunotherapy biomarker: utility for the oncology clinic. Ann. Oncol..

[bib7] Cooper L.A., Demicco E.G., Saltz J.H., Powell R.T., Rao A., Lazar A.J. (2018). PanCancer insights from the Cancer Genome Atlas: the pathologist's perspective. J. Pathol..

[bib8] Coudray N., Ocampo P.S., Sakellaropoulos T., Narula N., Snuderl M., Fenyö D., Moreira A.L., Razavian N., Tsirigos A. (2018). Classification and mutation prediction from non-small cell lung cancer histopathology images using deep learning. Nat. Med..

[bib9] Cristescu R., Mogg R., Ayers M., Albright A., Murphy E., Yearley J., Sher X., Liu X.Q., Lu H., Nebozhyn M. (2018). Pan-tumor genomic biomarkers for PD-1 checkpoint blockade-based immunotherapy. Science.

[bib10] Du X., Tang F., Liu M., Su J., Zhang Y., Wu W., Devenport M., Lazarski C.A., Zhang P., Wang X. (2018). A reappraisal of CTLA-4 checkpoint blockade in cancer immunotherapy. Cell Res..

[bib11] Eisenhauer E.A., Therasse P., Bogaerts J., Schwartz L.H., Sargent D., Ford R., Dancey J., Arbuck S., Gwyther S., Mooney M. (2009). New response evaluation criteria in solid tumours: revised RECIST guideline (Version 1.1). Eur. J. Cancer.

[bib12] Esteva A., Kuprel B., Novoa R.A., Ko J., Swetter S.M., Blau H.M., Thrun S. (2017). Dermatologist-level classification of skin cancer with deep neural networks. Nature.

[bib13] Ferris R.L., Blumenschein G., Fayette J., Guigay J., Colevas A.D., Licitra L., Harrington K., Kasper S., Vokes E.E., Even C. (2016). Nivolumab for recurrent squamous-cell carcinoma of the head and neck. N. Engl. J. Med..

[bib14] Fu Y., Jung A.W., Torne R.V., Gonzalez S., Vöhringer H., Shmatko A., Yates L.R., Jimenez-Linan M., Moore L., Gerstung M. (2020). Pan-cancer computational histopathology reveals mutations, tumor composition and prognosis. Nat. Cancer.

[bib15] Ganin Y., Ustinova E., Ajakan H., Germain P., Larochelle H., Laviolette F., Marchand M., Lempitsky V.S. (2016). Domain-adversarial training of neural networks. J. Mach. Learn. Res..

[bib16] He B., Dong D., She Y., Zhou C., Fang M., Zhu Y., Zhang H., Huang Z., Jiang T., Tian J., Chen C. (2020). Predicting response to immunotherapy in advanced non-small-cell lung cancer using tumor mutational burden radiomic biomarker. J. Immunother. Cancer.

[bib17] Hegde P.S., Chen D.S. (2020). Top 10 challenges in cancer immunotherapy. Immunity.

[bib18] Hellmann M.D., Ciuleanu T.-E., Pluzanski A., Lee J.S., Otterson G.A., Audigier-Valette C., Minenza E., Linardou H., Burgers S., Salman P. (2018). Nivolumab plus ipilimumab in lung cancer with a high tumor mutational burden. N. Engl. J. Med..

[bib19] Herbst R.S., Baas P., Kim D.W., Felip E., Pérez-Gracia J.L., Han J.Y., Molina J., Kim J.H., Arvis C.D., Ahn M.J. (2016). Pembrolizumab versus docetaxel for previously treated, Pd-L1-Positive, advanced non-small-cell lung cancer (Keynote-010): a randomised controlled trial. Lancet.

[bib20] Hugo W., Zaretsky J.M., Sun L., Song C., Moreno B.H., Hu-Lieskovan S., Berent-Maoz B., Pang J., Chmielowski B., Cherry G. (2016). Genomic and transcriptomic features of response to anti-PD-1 therapy in metastatic melanoma. Cell.

[bib21] Jain M.S., Massoud T.F. (2020). Predicting tumour mutational burden from histopathological images using multiscale deep learning. Nat. Mach. Intell..

[bib22] Jiang T., Shi J., Dong Z., Hou L., Zhao C., Li X., Mao B., Zhu W., Guo X., Zhang H. (2019). Genomic landscape and its correlations with tumor mutational burden, PD-L1 expression, and immune cells infiltration in Chinese lung squamous cell carcinoma. J. Hematol. Oncol..

[bib23] Kather J.N., Krisam J., Charoentong P., Luedde T., Herpel E., Weis C.-A., Gaiser T., Marx A., Valous N.A., Ferber D. (2019). Predicting survival from colorectal cancer histology slides using deep learning: a retrospective multicenter study. PLoS Med..

[bib24] Kather J.N., Pearson A.T., Halama N., Jäger D., Krause J., Loosen S.H., Marx A., Boor P., Tacke F., Neumann U.P. (2019). Deep learning can predict microsatellite instability directly from histology in gastrointestinal cancer. Nat. Med..

[bib25] Kingma D.P., Ba J. (2015).

[bib26] Koboldt D.C., Zhang Q., Larson D.E., Shen D., Mclellan M.D., Lin L., Miller C.A., Mardis E.R., Ding L., Wilson R.K. (2012). VarScan 2: somatic mutation and copy number alteration discovery in cancer by exome sequencing. Genome Res..

[bib27] Krizhevsky A., Sutskever I., Hinton G.E. (2017). ImageNet classification with deep convolutional neural networks. Commun. ACM.

[bib28] Loshchilov I., Hutter F. (2017). SGDR: stochastic gradient descent with warm restarts. arXiv.

[bib29] Lu M.Y., Chen T.Y., Williamson D.F.K., Zhao M., Shady M., Lipkova J., Mahmood F. (2021). AI-based pathology predicts origins for cancers of unknown primary. Nature.

[bib30] Marabelle A., Fakih M., Lopez J., Shah M., Shapira-Frommer R., Nakagawa K., Chung H.C., Kindler H.L., Lopez-Martin J.A., Miller W.H. (2020). Association of tumour mutational burden with outcomes in patients with advanced solid tumours treated with pembrolizumab: prospective biomarker analysis of the multicohort, open-label, phase 2 KEYNOTE-158 study. Lancet Oncol..

[bib31] Paszke A., Gross S., Massa F., Lerer A., Bradbury J., Chanan G., Killeen T., Lin Z., Gimelshein N., Antiga L. (2019). Proceedings of the 33rd International Conference on Neural Information Processing Systems.

[bib32] Paszke A., Gross S., Massa F., Lerer A., Bradbury J., Chanan G., Killeen T., Lin Z., Gimelshein N., Antiga L. (2019).

[bib33] Ribas A., Wolchok J.D. (2018). Cancer immunotherapy using checkpoint blockade. Science.

[bib34] Rizvi N.A., Hellmann M.D., Snyder A., Kvistborg P., Makarov V., Havel J.J., Lee W., Yuan J., Wong P., Ho T.S. (2015). Cancer immunology. Mutational landscape determines sensitivity to PD-1 blockade in non-small cell lung cancer. Science.

[bib35] Schmauch B., Romagnoni A., Pronier E., Saillard C., Maillé P., Calderaro J., Kamoun A., Sefta M., Toldo S., Zaslavskiy M. (2020). A deep learning model to predict RNA-Seq expression of tumours from whole slide images. Nat. Commun..

[bib36] Somasundaram R., Connelly T., Choi R., Choi H., Samarkina A., Li L., Gregorio E., Chen Y., Thakur R., Abdel-Mohsen M. (2021). Tumor-infiltrating mast cells are associated with resistance to anti-PD-1 therapy. Nat. Commun..

[bib37] Sun R., Limkin E.J., Vakalopoulou M., Dercle L., Champiat S., Han S.R., Verlingue L., Brandao D., Lancia A., Ammari S. (2018). A radiomics approach to assess tumour-infiltrating CD8 cells and response to anti-PD-1 or anti-PD-L1 immunotherapy: an imaging biomarker, retrospective multicohort study. Lancet Oncol..

[bib38] Szegedy C., Vanhoucke V., Ioffe S., Shlens J., Wojna Z. (2016). 2016 IEEE Conference on Computer Vision and Pattern Recognition (CVPR).

[bib39] Tan M., Le Q.V. (2019). EfficientNet: rethinking model scaling for convolutional neural networks. ArXiv.

[bib40] Xu H., Park S., Lee S.H., Hwang T.H. (2019).

[bib41] Yoo S.-Y., Park H.E., Kim J.H., Wen X., Jeong S., Cho N.-Y., Gwon H.G., Kim K., Lee H.S., Jeong S.-Y. (2020). Whole-slide image analysis reveals quantitative landscape of tumor-immune microenvironment in colorectal cancers. Clin. Cancer Res..

[bib42] Zappasodi R., Merghoub T., Wolchok J.D. (2018). Emerging concepts for immune checkpoint blockade-based combination therapies. Cancer Cell.

[bib43] Zhao Q., Adeli E., Pohl K.M. (2020). Training confounder-free deep learning models for medical applications. Nat. Commun..

