# Supplementary material for: Genopathomic profiling identifies signatures for immunotherapy response of lung adenocarcinoma via confounder-aware representation learning
Source: iScience. 2022 Oct 17;25(11):105382. doi: 10.1016/j.isci.2022.105382 (PMC9636035; doi:10.1016/j.isci.2022.105382)
Supplement: Document S1. Figures S1–S6 and Tables S1–S7 [file mmc1.pdf]

iScience, Volume *25*

Supplemental information

Genopathomic profiling identifies signatures for immunotherapy response of lung adenocarcinoma via confounder-aware representation learning

Jiajun Deng, Jiancheng Yang, Likun Hou, Junqi Wu, Yi He, Mengmeng Zhao, Bingbing Ni, Donglai Wei, Hanspeter Pfister, Caicun Zhou, Tao Jiang, Yunlang She, Chunyan Wu, and Chang Chen

Supplemental Figures and Tables

Figure S1. Confusion matrices in histology classification in the TCGA dataset (A, accuracy, 0.96) and SPH (B, accuracy, 0.81) dataset. Related to Figure 1B. The row indicates the predicted label, and the column indicates the actual label of each slide.

Figure S2. TMB prediction performance in patients with lung squamous cell carcinoma. Related to STAR Methods. (A) Independent validation of TMB prediction performance in lung squamous cell carcinoma by the PITER in the TCGA validation datasets. (B) Scatter plots of changes in tumor mutational burden value according to probability output from the PITER in the TCGA datasets. AUC, area under curve. TMB, tumor mutational burden.

Figure S3. Overall survival time predicted by the PITER in a subset of the TCGA validation dataset. Related to Figure 4.

Figure S4. Correlation heatmap among different components of the immune microenvironment. Related to Figure 4.

Figure S5. Flowchart of patient inclusion and exclusion. Related to Figure 1A. WSI, whole-slide images. LUAD, lung adenocarcinoma. LUSC, lung squamous cell carcinoma. TMB, tumor mutational burden.

Figure S6. The causal graphs of the procedure. Related to Figure 1B. The ideal causal graph (left) $w \rightarrow b \rightarrow o$ could not be implemented directly due to memory constraints. The sliding-window approach (middle) introduced confounder w, which could be a shortcut for learning. The proposed method (right), adversarial confounder suppression (AdvCS), eliminated the influence of confounding variables and reduced overfitting, thereby improving generalization performance.

Table S1. Baseline Characteristics of TCGA LUAD dataset. Related to Figure 1A.

Table S2. Baseline Characteristics of SPH LUAD TMB dataset. Related to Figure 1A.

Table S3. Baseline Characteristics of TCGA LUSC dataset. Related to Figure 1A.

Table S4. Baseline Characteristics of SPH LUSC TMB dataset. Related to Figure 1A.

Table S5. Baseline Characteristics of Immunotherapy dataset. Related to Figure 3.

Table S6. Multivariable Cox Regression Analysis for progression-free and overall survival in Immunotherapy dataset. Related to Figure 3.

Table S7. Design analysis on TCGA LUAD dataset. Related to Figure 1B and STAR Methods.

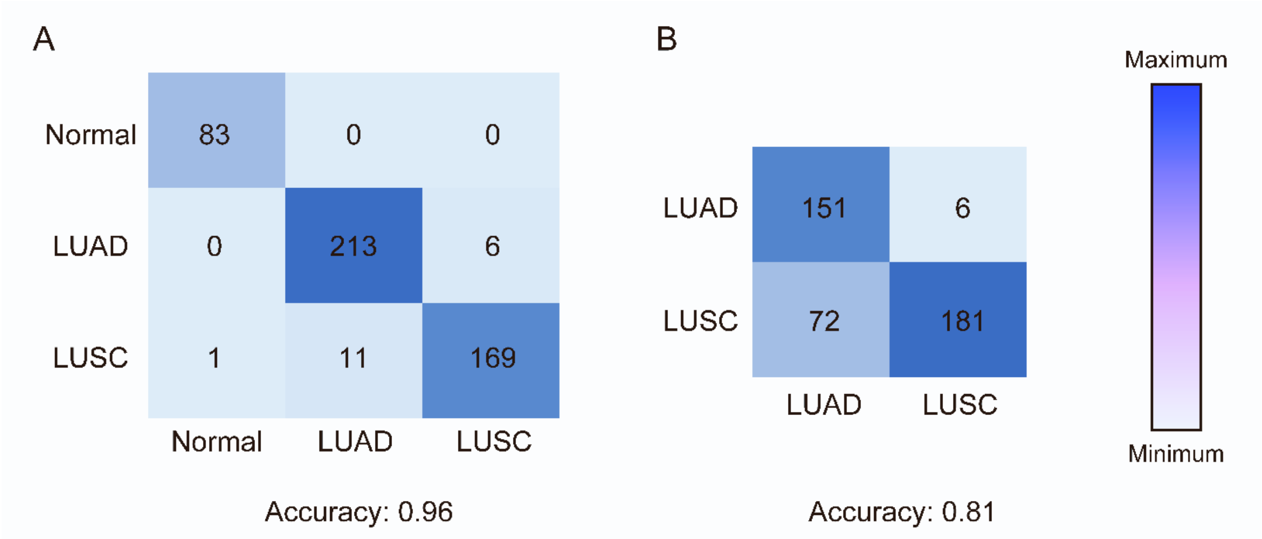

Figure S1. Confusion matrices in histology classification in the TCGA dataset (A, accuracy, 0.96) and SPH (B, accuracy, 0.81) dataset. The row indicates the predicted label, and the column indicates the actual label of each slide.

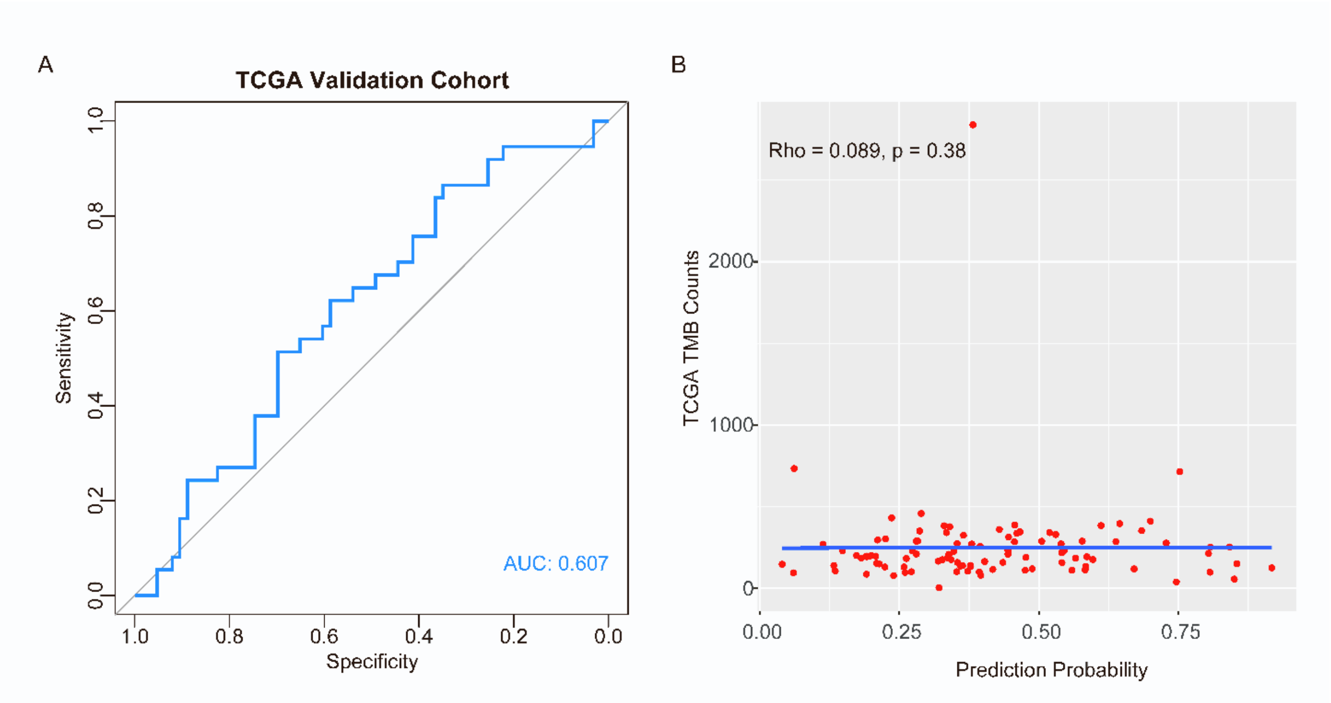

Figure S2. TMB prediction performance in patients with lung squamous cell carcinoma. (A) Independent validation of TMB prediction performance in lung squamous cell carcinoma by the PITER in the TCGA validation datasets. (B) Scatter plots of changes in tumor mutational burden value according to probability output from the PITER in the TCGA datasets. AUC, area under curve. TMB, tumor mutational burden.

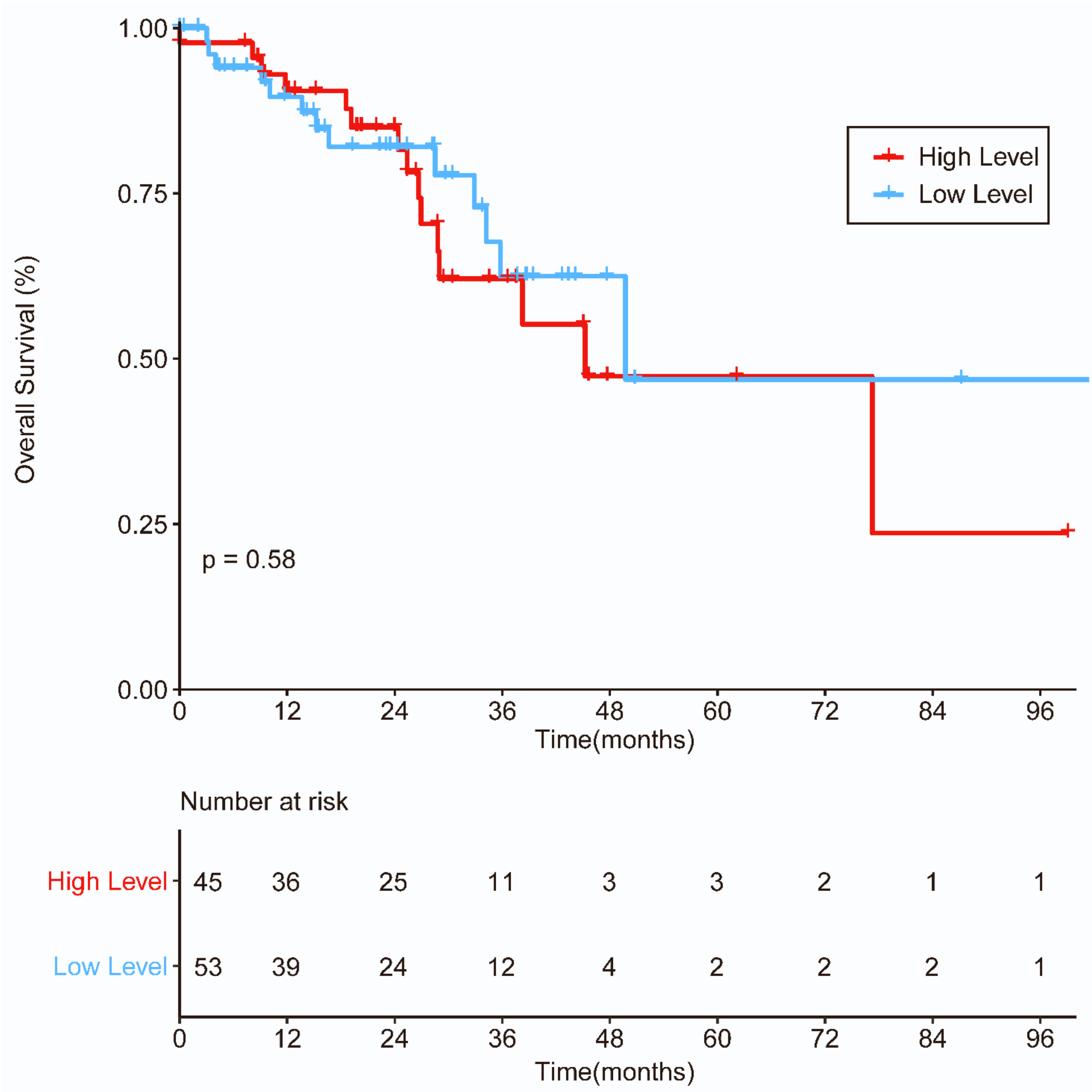

Figure S3. Overall survival time predicted by the PITER in a subset of the TCGA validation dataset.

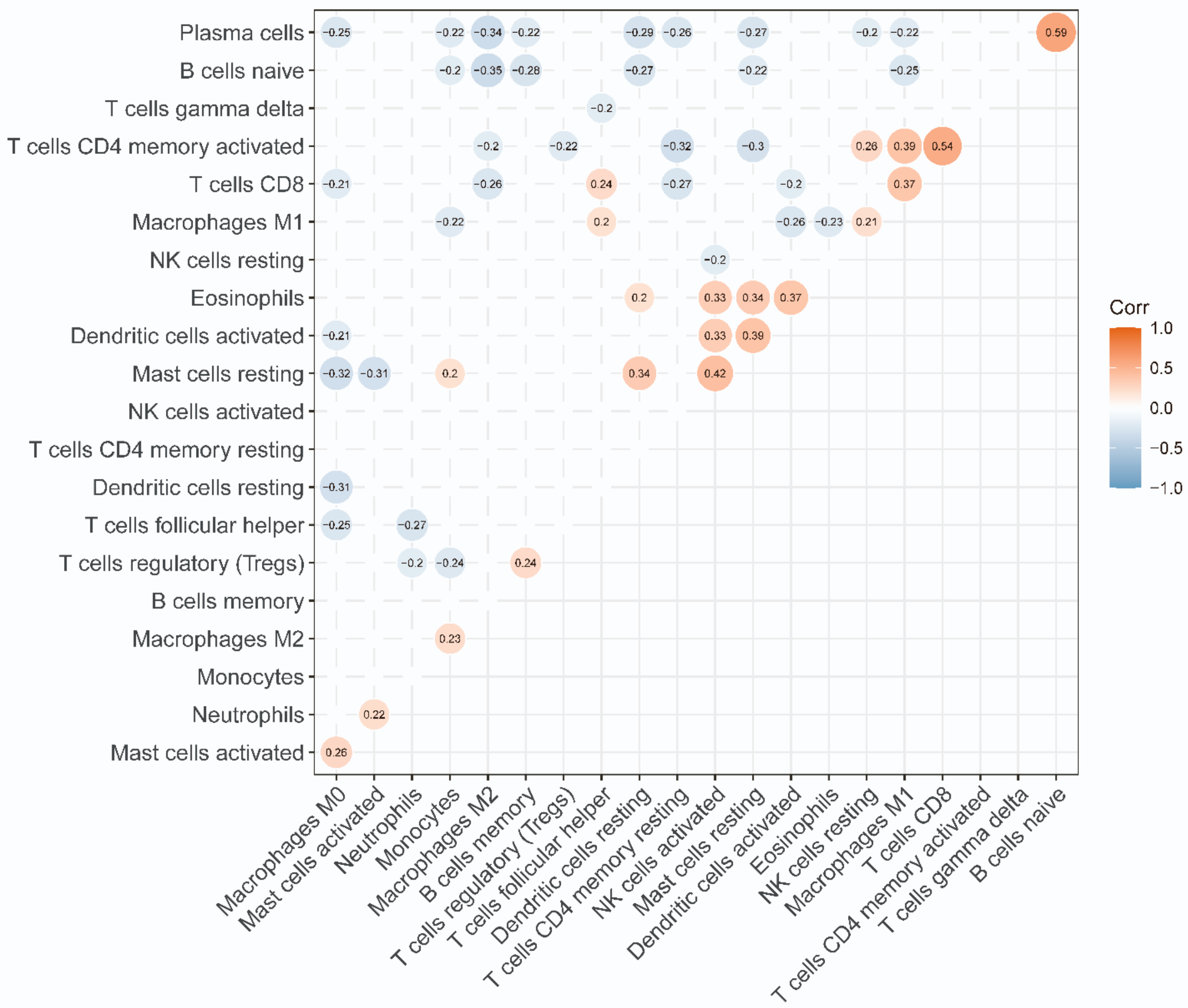

Figure S4. Correlation heatmap among different components of the immune microenvironment.

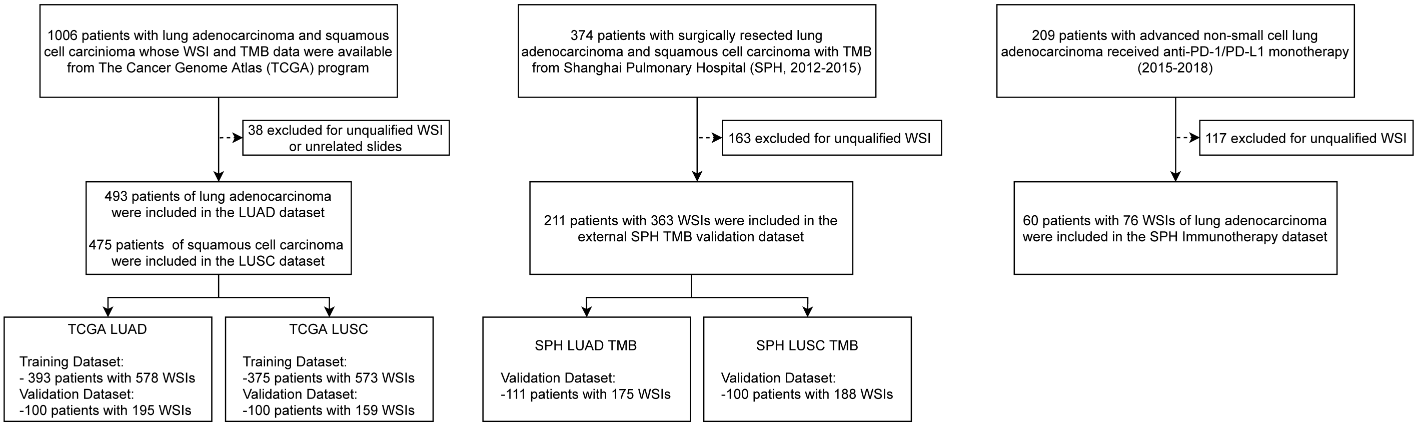

Figure S5. Flowchart of patient inclusion and exclusion. WSI, whole-slide images. LUAD, lung adenocarcinoma. LUSC, lung squamous cell carcinoma. TMB, tumor mutational burden.

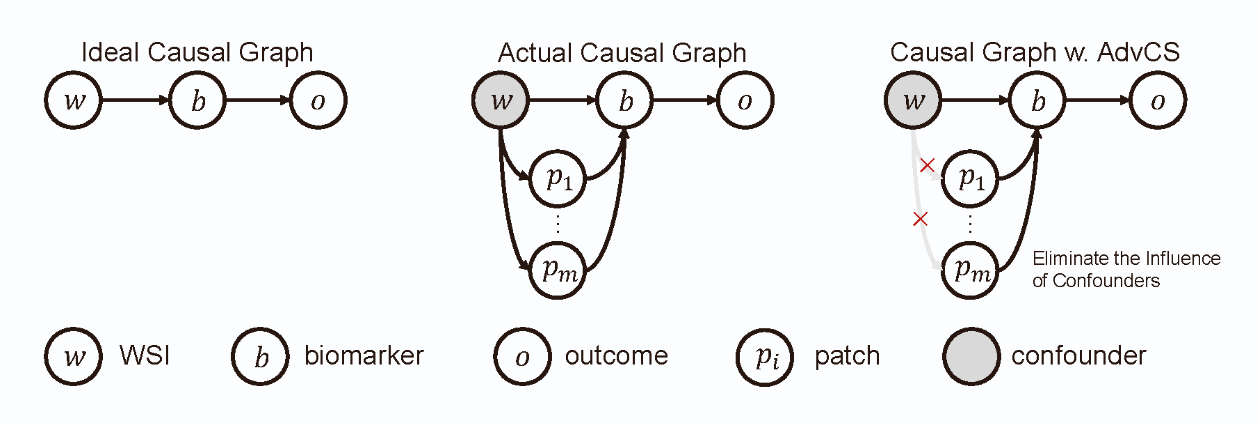

Figure S6. The causal graphs of the procedure. The ideal causal graph (left) $w \rightarrow b \rightarrow o$ could not be implemented directly due to memory constraints. The sliding-window approach (middle) introduced confounder w, which could be a shortcut for learning. The proposed method (right), adversarial confounder suppression (AdvCS), eliminated the influence of confounding variables and reduced overfitting, thereby improving generalization performance.

Table S1. Baseline Characteristics of TCGA LUAD dataset.

Variables	All (N=493)	Training Dataset (N=393)	Validation Dataset (N=100)	P Value
Age	63·3 ± 14·94	63·04 ± 15·96	64·31 ± 9·96	0·32
Sex				0·45
Male	263 (53·3)	213 (54·2)	50 (50)	
Female	230 (46·7)	180 (45·8)	50 (50)	
Stage				0·62
I	267 (54·2)	206 (52·4)	61 (61)	
II	114 (23·1)	95 (24·2)	19 (19)	
III	81 (16·4)	67 (17)	14 (14)	
IV	24 (4·9)	19 (4·8)	5 (5)	
Unknown	7 (1·4)	6 (1·5)	1 (1)	
TMB Counts	251·01 ± 273·65	248·1 ± 279·84	262·42 ± 248·83	0·64
Slides	773	578 (74·8)	195 (25·2)	
Patches	128725	99063 (77·0)	29662 (23·0)	

TCGA, the cancer genome atlas. LUAD, lung adenocarcinoma. TMB, tumor mutational burden.

Table S2. Baseline Characteristics of SPH LUAD TMB dataset.

Variables	All (N=111)	Low Level (N=98)	High Level (N=13)	P Value
Age	61·78 ± 0·82	62·01 ± 8·97	60·08 ± 5·41	0·28
Sex				**<0·01**
Male	49 (44·1)	38 (38·8)	11 (84·6)	
Female	62 (55·9)	60 (61·2)	2 (15·4)	
Stage				0·57
I	78 (70·3)	70 (71·4)	8 (61·5)	
II	10 (9)	8 (8·2)	2 (15·4)	
III	19 (17·1)	16 (16·3)	3 (23·1)	
IV	4 (3·6)	4 (4·1)	0 (0)	
EGFR Mutation				**<0·01**
No	41 (36·9)	31 (31·6)	10 (76·9)	
Yes	70 (63·1)	67 (68·4)	3 (23·1)	
TMB Value	0·36 ± 0·01	0·34 ± 0·11	0·49 ± 0·17	**<0·01**

LUAD, lung adenocarcinoma. TMB, tumor mutational burden.

Table S3. Baseline Characteristics of TCGA LUSC dataset.

Variables	All (N=475)	Training Dataset (N=375)	Validation Dataset (N=100)	P Value
Age	63·3 ± 14·94	65·88 ± 12·28	66·33 ± 13·58	0·75
Sex				0·85
Male	355 (74·7)	281 (74·9)	74 (74)	
Female	120 (25·3)	94 (25·1)	26 (26)	
Stage				0·16
I	230 (48·4)	178 (47·5)	52 (52)	
II	154 (32·4)	128 (34·1)	26 (26)	
III	80 (16·8)	59 (15·7)	21 (21)	
IV	7 (1·5)	7 (1·9)	0 (0)	
Unknown	4 (0·8)	3 (0·8)	1 (1)	
TMB Counts	251·01 ± 273·65	264·33 ± 211·85	246·38 ± 288·56	0·49
Slides	732	573 (78·3)	159 (21·7)	
Patches	137821	111611 (81·0)	26210 (19·0)	

TCGA, the cancer genome atlas. LUSC, lung squamous cell carcinoma. TMB, tumor mutational burden.

Table S4. Baseline Characteristics of SPH LUSC TMB dataset.

Variables	All (N=100)	Low Level (N=57)	High Level (N=43)	P Value
Age	63 ± 8·4	62·49 ± 9·28	63·67 ± 7·12	0·47
Sex				1
Male	96 (96)	55 (96·5)	41 (95·3)	
Female	4 (4)	2 (3·5)	2 (4·7)	
Smoke				0·17
Ever	30 (30)	14 (24·6)	16 (37·2)	
Never	70 (70)	43 (75·4)	27 (62·8)	
Stage				0·62
I	46 (46)	28 (49·1)	18 (41·9)	
II	32 (32)	16 (28·1)	16 (37·2)	
III	22 (22)	13 (22·8)	9 (20·9)	
TMB Value	0·48 ± 0·14	0·48 ± 0·15	0·48 ± 0·14	0·93

LUSC, lung squamous cell carcinoma. TMB, tumor mutational burden.

Table S5. Baseline Characteristics of Immunotherapy dataset.

Variables	All (N=60)	Low Level (N=15)	High Level (N=45)	P Value
Age	58 ± 9·86	57·47 ± 10·6	58·18 ± 9·73	0·81
Sex				**0·01**
Male	49 (81·7)	9 (60)	40 (88·9)	
Female	11 (18·3)	6 (40)	5 (11·1)	
Smoke				0·13
Never	30 (50)	10 (66·7)	20 (44·4)	
Ever	30 (50)	5 (33·3)	25 (55·6)	
Stage				0·32
III	5 (8·3)	0 (0)	5 (11·1)	
IV	55 (91·7)	15 (100)	40 (88·9)	
Mutation				1·00
No	46 (76·7)	11 (73·3)	35 (77·8)	
Yes	14 (23·3)	4 (26·7)	10 (22·2)	
PITER score	0·43 ± 0·25	0·13 ± 0·05	0·53 ± 0·21	**<0·01**

The grouping of low and high levels is stratified by our proposed method at the cutoff at 0·23. Continuous variables are reported as mean ± standard deviation. Categorical variables are reported as count (percentage). PITER score, the output score of the genopathomic biomarker for immunotherapy response.

Table S6. Multivariable Cox Regression Analysis for progression-free and overall survival in Immunotherapy dataset.

**Variables**	**PFS**		**OS**	
	**HR (95% CI)**	**P Value**	**HR (95% CI)**	**P Value**
Age	1·01 (0·97 - 1·06)	0·51	0·95 (0·91 - 1·00)	0·07
Sex (Male)	1·16 (0·43 - 3·14)	0·77	0·94 (0·20 - 4·51)	0·94
Smoke (Never)	0·57 (0·29 - 1·11)	0·10	0·88 (0·19 - 4·06)	0·87
Stage (III)	0·60 (0·20 - 1·76)	0·35	0·91 (0·10 - 8·27)	0·93
Mutation (Wild type)	0·79 (0·31 - 1·98)	0·61	1·02 (0·25 - 4·07)	0·98
PITER score	0·44 (0·21 - 0·92)	**0·03**	0·32 (0·10 - 0·99)	**0·04**

Variables in the bracket were set as reference. PFS, progression-free survival. OS, overall survival. HR, hazard ratio. CI, confidence interval. PITER score, the output score of the genopathomic biomarker for immunotherapy response.

Table S7. Design analysis on TCGA LUAD dataset.

Design \ Backbone	Inception-V3 (AUC)	EfficientNet-B0 (AUC)
Plain	0·767	0·782
+ Stain Normalization	0·806	0·822
+ AdvCS	0·831	0·853

TCGA, the cancer genome atlas. LUAD, lung adenocarcinoma. AdvCS, adversarial confounder suppression (proposed). AUC, area under receiver operating characteristic curve.
